# Mendelian Randomization Analysis Reveals Causal Associations Between Beverages and Irritable Bowel Syndrome: Alcohol, but Not Others

**DOI:** 10.1002/fsn3.70761

**Published:** 2025-07-31

**Authors:** Ruiqing Yuan, Yajing Zhou, Yujie Ren, Xiaohua Hou, Siran Zhu

**Affiliations:** ^1^ Division of Gastroenterology Union Hospital, Tongji Medical College, Huazhong University of Science and Technology Wuhan China; ^2^ Department of Respiratory and Critical Care Medicine National Clinical Research Center of Respiratory Disease, Key Laboratory of Pulmonary Diseases of Health Ministry, Tongji Hospital, Tongji Medical College and State Key Laboratory of Pulm, Huazhong University of Science and Technology Wuhan China

**Keywords:** alcohol intake, gut microbiota, irritable bowel syndrome, mendelian randomization, psychiatric disorders

## Abstract

Observational studies have suggested that beverage intake could have potential connections with irritable bowel syndrome (IBS). However, the association patterns and the underlying mechanisms remain unknown. The study identified potential causal relationships between alcohol intake and IBS, showing positive associations for alcohol intake frequency (odds ratio = 1.18, 95% confidence interval = 1.09–1.26; *p* < 0.001). Psychiatric disorders were found to play mediative roles with mediation effects of 25.22%, 45.77%, and 12.10% for depression (broad), major depression disease, and attention deficit hyperactivity disorder, respectively. These findings suggest that reducing alcohol intake may help prevent IBS, especially in individuals with psychiatric conditions.

## Graphical abstract

Genetic evidence links alcohol to increased IBS risk, mediated by psychiatric factors (25%–45%), informing new management strategies.

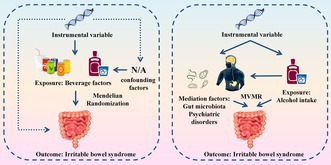

**Abbreviations**
ADHD: attention deficit hyperactivity disorder
ASD: autism spectrum disorder
DHQ: digestive health questionnaire
FDR: false discovery rate
GSCAN: GWAS & Sequencing Consortium of Alcohol and Nicotine use
GWAS: genome‐wide association studies
IBS: irritable bowel syndrome
IV: instrumental variable
IVW: inverse‐variance‐weighted
LD: linkage disequilibrium
MDD: major depressive disorder
MR: Mendelian randomization
MRC‐IEU: MRC Integrative Epidemiology Unit
MR‐PRESSO: MR pleiotropy residual sum and outlier
MVMR: multivariable Mendelian randomization
OR: odds ratio
PPD: postpartum depression
PTSD: post‐traumatic stress disorder
SNP: single nucleotide polymorphis

## 1 Introduction

Irritable bowel syndrome (IBS), a chronic functional gastrointestinal disorder characterized by abdominal symptoms and altered bowel habits, impacts 7% to 21% of the global population (Chey et al. 2015; Spiller 2021). The intricate pathological mechanisms underlying IBS remain elusive, with the gut‐brain axis, visceral hypersensitivity, impaired barrier function, and altered gut motility being among the theories proposed to explain its pathogenesis (Ford et al. 2020). Dietary factors play dual roles in IBS: triggers for symptoms but also tools for therapy (El‐Salhy et al. 2019; Pasta et al. 2024). Management of specific dietary factors (e.g., Gluten) has been recognized as an effective possibility for treating IBS (Rej et al. 2022). However, the relationship between beverages, especially alcohol, and IBS remains unclear.

Alcohol is well‐known to have effects on the motility, absorption, and permeability of the gastrointestinal tract (Cozma‐Petruţ et al. 2017; Pohl et al. 2021). However, it is still unknown whether alcohol intake has a causal relationship with IBS. An observational study of women aged 18–48 years with IBS and healthy controls has revealed a positive correlation between high alcohol intake and the gastrointestinal symptoms of IBS patients (Reding et al. 2013). Additionally, another study among 123 female IBS patients with a lower Somatic Symptom Score has indicated that high alcohol intake heightens the risk of IBS (Halder et al. 2006). However, there are studies drawing contrasting conclusions. An investigation has found no significant link between high alcohol intake and IBS (Locke III et al. 2000), while other studies have suggested high alcohol intake is a protective factor for IBS (Yuan et al. 2023).

Previous research has demonstrated that excessive alcohol intake can lead to psychiatric disorders, including depression and anxiety (Jakubczyk et al. 2018; Xu et al. 2022). Furthermore, a meta‐analysis exploring the relationship between alcohol use disorder and major depressive disorder (MDD) concluded that alcohol use disorder increases the risk of developing MDD and vice versa (Boden and Fergusson 2011). Notably, psychiatric disorders frequently coexist with IBS (Fond et al. 2014), with a Mendelian randomization study highlighting the causal association between a broad spectrum of psychiatric conditions, such as depression, MDD, anxiety disorders, post‐traumatic stress disorder (PTSD), and schizophrenia, and IBS (Zhang et al. 2024). These findings underscore the intricate interplay between alcohol intake, mental health, and the gastrointestinal system, but the specific mechanism remains to be discussed.

Alcohol intake has also been reported to induce gut microbiota dysbiosis, specifically characterized by a significant decrease in probiotics and an increase in opportunistic pathogens (Leclercq et al. 2019). For example, alcohol use disorder can lead to decreased levels of anti‐inflammatory bacteria, including *Faecalibacterium prausnitzii* and *Bifidobacterium*, and an increased abundance of *Proteobacteria* (Wang et al. 2020). Previous studies have shown that the abundance of probiotics and pathogens is associated with IBS. A bidirectional Mendelian analysis between 196 bacterial traits and IBS found that several changes in gut microbiota can influence the incidence of IBS (Liu et al. 2023). The above evidence indicated that altered gut microbiota might play a part in the association between alcohol intake and IBS.

In this study, we aimed to clarify the associations between alcohol intake and IBS by performing two‐sample Mendelian analyses. Furthermore, we aimed to identify the possible mediating roles of psychiatric disorders and gut microbiota.

## 2 Materials and Methods

### 2.1 Study Design

**FIGURE 1:**
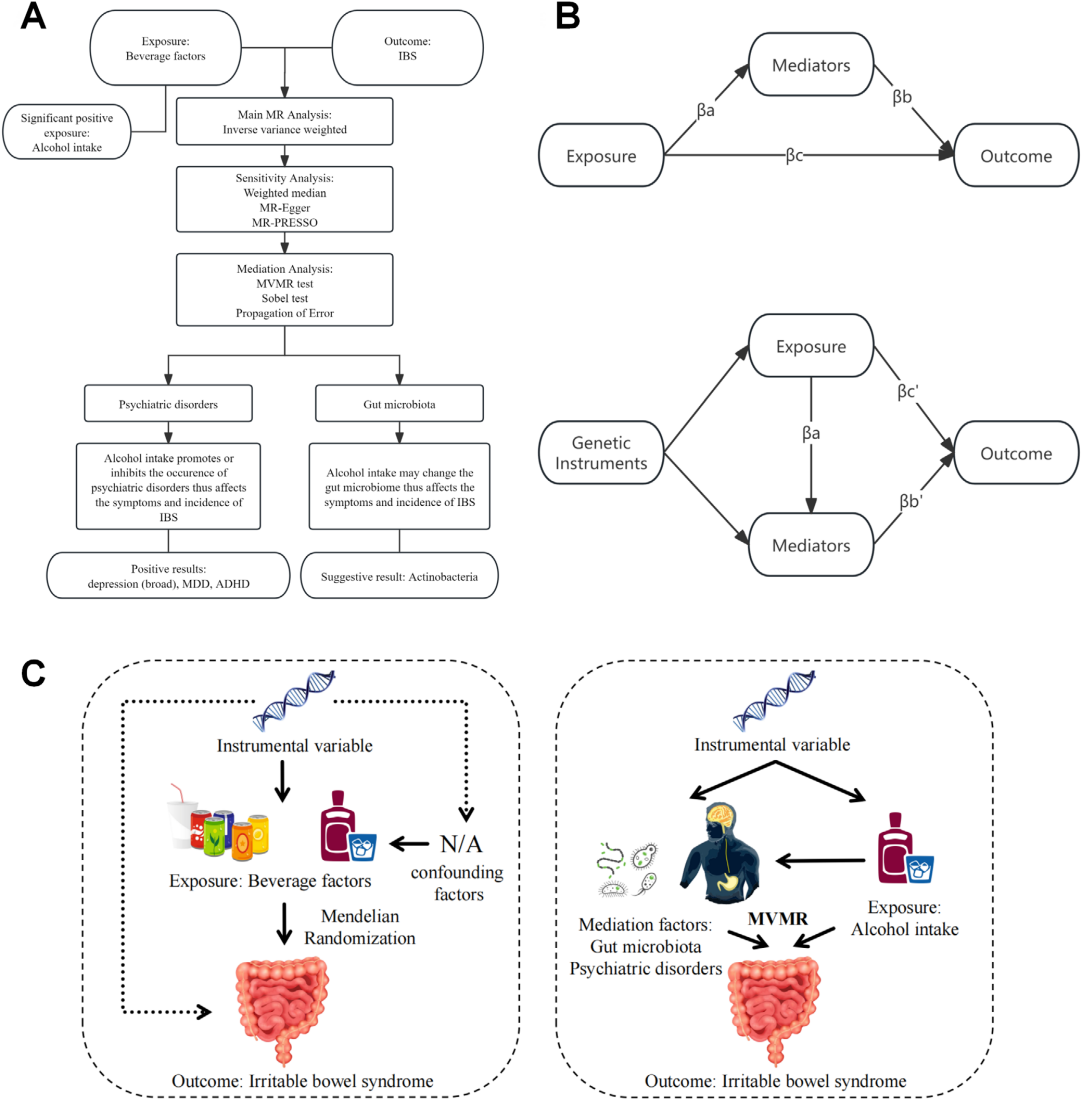
(A) The process and design of our study. (B) The process of the mediation analysis. *βa*, the effect of exposures on mediators; *βb*, the effect of mediators on outcomes; *βc*, the effect of exposures on outcomes; *βc*′, the effect of exposures outcomes after MVMR adjustment; *βb*′, the effect of mediators on outcomes after MVMR adjustment; ADHD, attention deficit hyperactivity disorder; IBS, irritable bowel syndrome; MDD, major depression disease; MR, Mendelian randomization; MVMR, Multivariable Mendelian randomization. (C) The graphical abstract of our study.

Mendelian randomization (MR) analyses were performed to investigate the causal effects of alcohol intake on IBS. To be a valid instrument for causal inference in MR studies, genetic variations should meet three fundamental assumptions: (1) the genetic instruments are supposed to have direct associations with exposure; (2) the genetic instruments are supposed to be unrelated to the outcome and independent of any unknown or known confounding factors; (3) exposures of interest mediate the effects of instrumental variables (IVs) on the outcomes (Gu et al. 2023). The design of our study is presented in Figure 1A.

### 2.2 Data Sources

Datasets used in our study were shown in Table S1.

#### 2.2.1 Irritable Bowel Syndrome

Genetic instruments for IBS were generated from a genome‐wide association study (GWAS) project including 53,400 cases and 433,201 controls of the European population (Eijsbouts et al. 2021). The inclusion criteria were:

(1) Patients' digestive health questionnaire (DHQ) met Rome III diagnostic criteria without other explanation for their symptoms. (2) The patients had been diagnosed by DHQ self‐reports. (3) The patients had been diagnosed with IBS by gastrointestinal doctors.

#### 2.2.2 Beverage Factors and Alcohol Intake

Genetic instruments of beverage factors, including water intake, milk intake, coffee intake, honey intake, orange juice intake, pure fruit/vegetable juice intake, grapefruit juice intake, and yogurt intake, were collected from the MRC Integrative Epidemiology Unit (MRC‐IEU) (Collins 2012). Genetic instruments associated with alcohol intake frequency were collected from the MRC‐IEU (Zhu et al. 2023). Genetic instruments for the amount of alcohol drink were collected from meta‐analyses of genome‐wide association studies conducted by the GWAS & Sequencing Consortium of Alcohol and Nicotine Use (GSCAN) (Liu et al. 2019).

#### 2.2.3 Psychiatric Diseases and Gut Microbiota

Genetic instruments of psychiatric diseases, including MDD, postpartum depression (PPD), attention deficit hyperactivity disorder (ADHD), PTSD, panic, schizophrenia, autism spectrum disorder (ASD), bipolar disorder, and Tourette syndrome, were collected from the Psychiatric Genomics Consortium (Logue et al. 2015). The statistical data on depression (broad) were obtained from a meta‐analysis comprising 246,363 cases and 561,190 controls (Howard et al. 2019). Genetic instruments of human gut microbiota were collected from the MiBioGen Study, which included 14,306 participants (Kurilshikov et al. 2021).

### 2.3 Selection of Single Nucleotide Polymorphism (SNP)

In order to ensure the statistical power of IVs, some of the thresholds for *p* value had been modified. The SNPs should meet the criterion of *p* < 5e‐06 for the exposures of beverage factors (except for *alcohol intake frequency, p* < 5e‐08). We selected the SNPs for human gut microbiota, psychiatric diseases, and IBS at *p* < 5e‐06.

Linkage disequilibrium (LD) indicates that genetic variations in the genome tend to be co‐inherited, resulting in a greater likelihood that alleles from two or more loci will co‐occur on a single chromosome than if they occur randomly (Gui et al. 2023). To obtain IVs from independent loci, we set the threshold for LD at *r* ^2^ < 0.001 and kb > 10,000. Here, “kb” represented the extent of regional LD, while the evaluation of “*r* ^2^” spanned from 0 to 1. An *r* ^2^ value of 1 indicates complete LD between the two SNPs, whereas an *r* ^2^ value of 0 signifies complete linkage equilibrium, where the assignment of the two SNPs is entirely random (Rizig et al. 2023).

We extracted the information on effects, other allele, and effect size (*β* value, standard error, *p* value) for each SNP. F‐statistics were conducted to evaluate the strength of the IVs. The IV is regarded as weak when the *F* value is less than 10 (Salehi Nowbandegani et al. 2023). The computational formulas are:

$$R^{2}=2\times \mathrm{MAF}\times \left(1-\mathrm{MAF}\right)\times \beta^{2}$$

$$F=R^{2}\;\left(n–k–1\right)/k\;\left(1–R^{2}\right)$$

“MAF” is the minor allele frequency of SNPs used as IVs, “*k*” is the number of IVs employed, and “*n*” is the sample size. *β* represents the allele effect size, and SD represents the standard deviation.

### 2.4 Statistical Analysis

All analyses and graph plotting were performed in R (version 4.3.3). In this study, *p* < 0.05 was considered statistically significant.

#### 2.4.1 Mendelian Randomization

Three distinct methods were employed for statistical analyses: inverse variance weighting (IVW), MR‐Egger regression, and weighted mode. Among these, IVW served as our primary analytical tool, entailing the consolidation of Wald estimates derived from individual genetic instruments, leveraging summary genotype data to arrive at an overarching estimation. These methods were also prevalent in most MR analysis studies (Burgess et al. 2013), underscoring their robustness. In the context of multiple comparisons, *p* values were corrected by a Benjamini–Hochberg false discovery rate (FDR) correction in order to mitigate false‐positive risks.

#### 2.4.2 Sensitivity Analysis

The heterogeneity of SNPs was investigated through the IVW and MR‐Egger methods, and the presence of heterogeneity is considered if *p* < 0.05 (Richardson et al. 2023). The MR‐Egger regression test was utilized to assess pleiotropy, which suggested the IVs could influence the outcome through other factors. The MR pleiotropy residual sum and outlier (MR‐PRESSO) test was used to exclude outliers. Furthermore, the “leave‐one‐out” method was used for a sensitivity analysis to assess outliers. It involves systematically removing the results associated with individual SNPs, evaluating whether they are outliers, and observing the stability of the results after removing each SNP (Cortez Cardoso Penha et al. 2023). Funnel plots were featured to assess the symmetry of SNPs and to show whether the results were credible.

#### 2.4.3 Mediation Analysis

A two‐step, two‐sample MR analysis was performed to investigate the possible mediation factors. Then, multi‐variable MR (MVMR) studies were tested to investigate their direct effects on IBS after mutual adjustment (Zhang et al. 2024). The proportion of mediators was calculated through the Propagation of Error method (Sanderson 2021). The Sobel test was employed to evaluate mediation effects when the GWAS summary data was not fully available and MVMR could not be conducted (Zhang et al. 2024). The process of the mediation analysis is represented in Figure 1B. *βa* represents the effect of exposures on mediators. *βb* represents the effect of mediators on outcomes. *βc* represents the effect of exposures on outcomes, while *βb*′ and *βc*′ represent the effect of exposures and mediators on outcomes after MVMR adjustment.

## 3 Results

### 3.1 Causal Associations Between Risk of IBS and Beverage Factors

The F‐statistics for the 10 beverage factors were above 10, underscoring their statistical significance. The MR results of the associations between all 10 beverage factors and IBS using the IVW method are presented in Table 1.

**TABLE 1:** Effect estimates of the associations of IBS with beverage factors.

Exposure	GWAS.ID	nSNP	*p*	*p*_adj_	Significance	OR	95% CI
Drinks per week	—	91	1.14E‐04	5.70E‐04	*	1.26	1.12–1.42
Alcohol intake frequency	ukb‐b‐5779	79	1.10E‐05	1.10E‐04	*	1.18	1.09–1.26
Water intake	ukb‐b‐14898	140	0.03	0.10	n.s.	1.16	1.01–1.34
Milk intake	ebi‐a‐GCST90096916	9	0.23	0.38	n.s.	0.21	0.02–2.65
Honey intake	ukb‐b‐6066	39	0.79	0.79	n.s.	0.98	0.82–1.17
Coffee intake	ukb‐b‐5237	39	0.31	0.39	n.s.	0.87	0.67–1.13
Orange juice intake	ukb‐b‐2763	12	0.44	0.49	n.s.	0.92	0.74–1.14
Pure fruit/vegetable juice intake	ukb‐b‐337	8	0.14	0.28	n.s.	1.34	0.91–1.97
Grapefruit juice intake	ukb‐b‐14351	19	0.28	0.39	n.s.	0.80	0.53–1.21
Yogurt intake	ukb‐b‐7753	9	0.06	0.15	n.s.	0.80	0.63–1.01

*Note:* *p*_adj_, *p* value after FDR correction. Drinks per week: the amount of alcohol intake per week. Alcohol intake frequency: the frequency of alcohol intake per week.

Abbreviations: 95% CI, 95% confidence interval; *, Significant; NSNP, the number of single nucleotide polymorphisms; n.s., Not significant; OR, odds ratios.

The results of MR analysis indicated that the frequency of alcohol intake patterns (including alcohol intake frequency, *p* = 1.10e‐05, *p*_adj_ = 1.10e‐04, odds ratio (OR) = 1.18; and drinks per week, *p* = 1.14e‐04, *p*_adj_ = 5.70e‐04, OR = 1.26) has a possible positive causal relationship with the risk of IBS.

**FIGURE 2:**
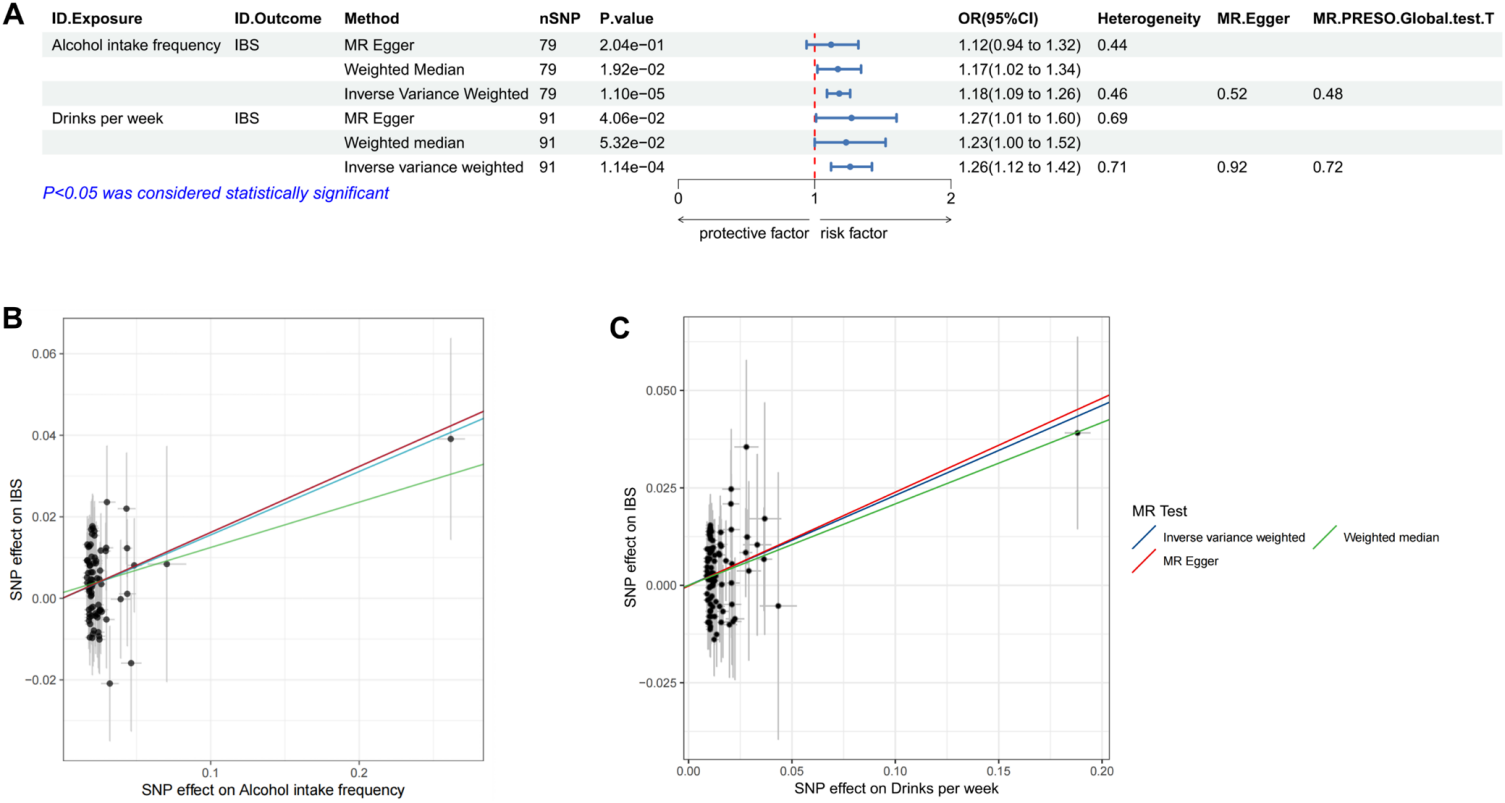
(A) The forest map of the association between alcohol and IBS. (B, C) Scatter plots of MR results. CI, confidence interval; OR, odds ratio; SNP, single nucleotide polymorphism.

Through additional experiments focusing specifically on alcohol intake and its impact on IBS risk, the results showed that alcohol intake frequency (IVW, *p* = 1.10e‐05, OR = 1.12; weighted median, *p* = 1.92e‐02, OR = 1.17) positively correlated with IBS through IVW and weighted median methods (Figure 2A,B, Figures S1 and S2). At the same time, drinks per week (IVW, *p* = 1.14e‐04, OR = 1.26; MR‐Egger, *p* = 4.06e‐02, OR = 1.27) showed significant associations with IBS through IVW and MR‐Egger methods (Figure 2A,C, Figures S3 and S4).

### 3.2 Mediation Effect of Gut Microbiota

In 193 bacterial traits, 7 bacterial traits were found to be related to IBS (Table S2). Eight bacterial traits were found to have a reverse relationship with IBS (Table S3). No overlapping bacterial trait was found between the forward and reverse researches. For alcohol intake, “alcohol intake frequency” was used as our exposure. As a result, 21 bacterial traits were found to be significantly related to alcohol intake (Table S4).

However, after applying FDR correction for multiple testing, we did not identify any gut microbiota as potential mediating factors.

### 3.3 Mediation Effect of Psychiatric Disorders

**FIGURE 3:**
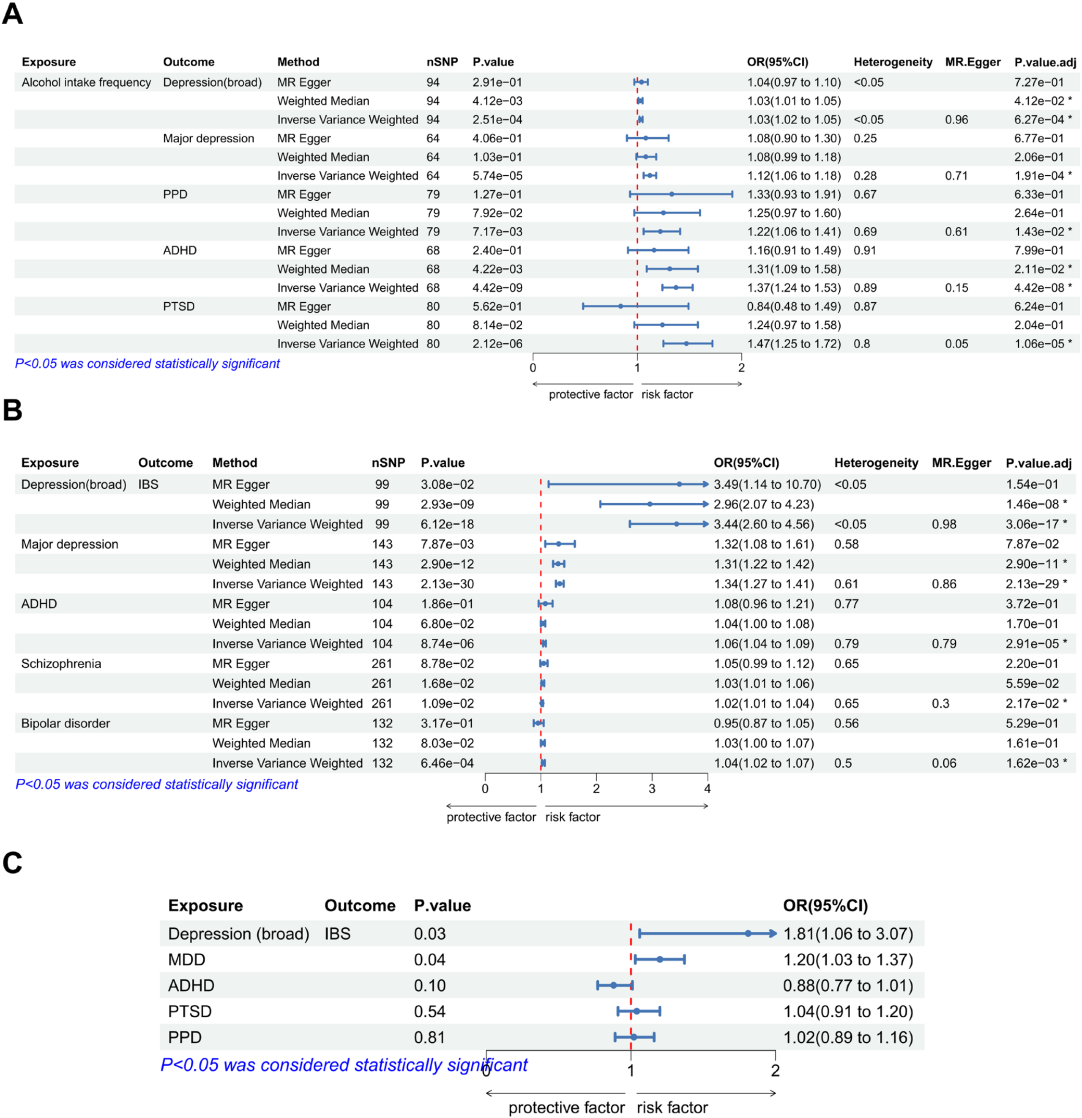
(A) Two‐sample MR analysis between alcohol intake frequency and psychiatric disorders. (B) Two‐sample MR analysis between psychiatric disorders and IBS. (C) Results of the MVMR test. ADHD, attention deficit hyperactivity disorder; ASD, autism spectrum disorder; CI, confidence interval; MVMR, multivariable Mendelian randomization; OR, odds ratio; *p*_adj_, *p* value after FDR correction; PPD, postpartum depression; PTSD, post‐traumatic stress disorder; SNP, single nucleotide polymorphism.

Our results indicated that alcohol intake could be a risk factor for psychiatric disorders (depression (broad): *p* = 2.51e‐04, *p*_adj_ = 6.27e‐04, OR = 1.03; MDD: *p* = 5.74e‐05, *p*_adj_ = 1.91e‐04, OR = 1.12; PPD: *p* = 7.17e‐03, *p*_adj_ = 1.43e‐02, OR = 1.22; ADHD: *p* = 4.42e‐09, *p*_adj_ = 4.42e‐08, OR = 1.37; PTSD: *p* = 2.12e‐06, *p*_adj_ = 1.06e‐05, OR = 1.47). Meanwhile, several psychiatric disorders were found to be the risk factors for IBS (depression (broad): *p* = 6.12e‐18, *p*_adj_ = 3.06e‐17, OR = 3.44; MDD: *p* = 2.13e‐30, *p*_adj_ = 2.13e‐29, OR = 1.32; ADHD: *p* = 8.74e‐06, *p*_adj_ = 2.91e‐05, OR = 1.06; schizophrenia: *p* = 1.09e‐02, *p*_adj_ = 2.17e‐02, OR = 1.02; bipolar disorder: *p* = 6.46e‐04, *p*_adj_ = 1.62e‐03, OR = 1.04). The IVW method was used as the primary analytical tool, and we found that depression (broad), MDD, and ADHD might be potential mediators (Figure 3A,B).

The MVMR method was used to estimate the effects of psychiatric disorders on IBS. Depression (broad) (*p* = 2.84e‐02, OR = 3.92) and MDD (*p* = 3.50e‐02, OR = 1.51) were still found to have direct positive effects on IBS (Figure 3C).

Then the MVMR method was utilized to adjust the influence of psychiatric disorders on the effect of alcohol intake. We found a significant attenuation in the effect of genetically predicted alcohol on IBS after the adjustment of depression (broad) and MDD, resulting in *βc*′ = 0.082 for depression (broad) adjustment and *βc*′ = −0.009 for MDD adjustment. However, we did not find significant results for ADHD, PTSD, and PPD via the MVMR method. As is shown in Table 2.

**TABLE 2:** The results of MR mediation analysis. *βa* represents the effect of exposures on mediators. *βb* represents the effect of mediators on outcomes. *βc* represents the effect of exposures on outcomes. While *βb*′ and *βc*′ represent the effect of exposures and mediators on outcomes after MVMR adjustment, respectively.

Exposure	Outcome	Mediator	*βc*	*βa*	*βb*	*βc*′	*βb*′	Sobel test	Mediated proportion (%)
			Effect size (95% CI)	Effect size (95% CI)	Effect size (95% CI)	Effect size (95% CI)	Effect size (95% CI)	*p*	
Alcohol intake frequency	IBS	Depression (broad)	0.162 (0.090–0.234)	0.033 (0.015–0.050)	0.42 (0.321–0.519)	0.082 (0.019–0.144)	1.238 (0.911–1.565)	**0.018**	25.22%^a^
		MDD	0.162 (0.090–0.234)	0.111 (0.057–0.165)	0.293 (0.243–0.343)	−0.009 (−0.105–0.087)	0.668 (0.403–0.933)	**< 0.01**	45.77%^a^
		ADHD	0.162 (0.090–0.234)	0.318 (0.212–0.424)	0.062 (0.034–0.089)	0.141 (0.033–0.249)	0.084 (−0.039–0.207)	**< 0.01**	12.10%^b^
		PTSD	0.162 (0.090–0.234)	0.385 (0.226–0.544)	0.002 (−0.041–0.045)	0.114 (0.021–0.207)	−0.017 (−0.124–0.091)	> 0.05	NA
		PPD	0.162 (0.090–0.234)	0.198 (0.054–0.342)	−0.008 (−0.028–0.014)	0.113 (0.020–0.206)	−0.01 (−0.132–0.111)	> 0.05	NA

*Note:* The results of the MVMR test: *p*, the *p* values after the adjustment are less than 0.05; *F*, the *p* values after the adjustment are more than 0.05 or unavailable result.

Abbreviation: NA, not available.

^a^ Results using the Propagation of Error method.

^b^ Results using the Sobel test.

Afterwards, we found that depression (broad) and MDD mediated 25.22% and 45.77% of the effects of alcohol intake on IBS. Sobel tests were performed instead, which indicated that ADHD has a significant mediation effect at a threshold of *p* < 0.05, which mediated 12.10% of the effects of alcohol intake on IBS.

## 4 Discussion

In this study, we found positive causal associations between alcohol intake and IBS. Furthermore, our findings indicated that depression (broad), MDD, and ADHD played mediating roles in the causal associations.

The evidence for the association between alcohol and IBS is contradictory. According to a meta‐analysis that summarized 1963 papers, one‐third of these studies suggested that high alcohol intake promotes the occurrence and development of IBS, while the rest indicated that there were protective effects or no effects (Creed 2019). Our study provided higher‐level statistical evidence for the possible role of alcohol intake as a risk factor for IBS through Mendelian Randomization analysis.

Previous studies have found some evidence indicating that a high‐level consumption of alcohol could damage intestinal function (Reding et al. 2013; Halder et al. 2006). Early studies in animals and patients already suggested that alcohol intake can disrupt the intestinal epithelial barrier, resulting in increased gut permeability (Stärkel et al. 2018). Oxidative stress plays a critical role in alcohol‐induced epithelial barrier disruption (Zhong et al. 2010), where the metabolite acetaldehyde can disrupt the epithelial tight junction integrity (Rao 2008). Furthermore, alcohol can have direct effects on the gastrointestinal tract, affecting the mucosa and altering orocecal transit times (Addolorato et al. 1997).

Our results did not identify any significant gut microbiota as mediating factors in the alcohol‐IBS association. However, these findings do not exclude the potential role of gut microbiota in this process. A study has suggested gut microbiota may represent implausible exposures. Some genetic predictors of gut microbiota are located in highly pleiotropic gene regions, such as the ABO gene region (Burgess et al. 2024). This suggests that the genetic predictors of gut microbiota may not necessarily serve as valid instrumental variables. The potential mediating role of gut microbiota in alcohol‐induced IBS pathogenesis needs further investigation.

Previous epidemiologic studies have found that alcohol can cause disruption of gut microbiota, which is the reduction of the abundance of probiotics and an increase of pernicious bacteria (Wang et al. 2020). Alcohol is widely believed to be able to alter the abundance of *Lactobacillus*, *Bifidobacterium*, *Klebsiella*, and other flora in the intestines, which are closely related to intestinal symptoms (Jew and Hsu 2023). *Lactobacillus* and *Bifidobacterium* species encode alcohol dehydrogenase and acetaldehyde dehydrogenase, which mediate alcohol and acetaldehyde metabolism (Jung et al. 2021). Alcohol‐related dysbiosis also affects the gut metabolome, including a decrease of short‐chain fatty acids (SCFAs), amino acids, and bile acids (Pohl et al. 2021), which can lead to impaired intestinal epithelial mucosal barrier and immune function, and can aggravate abdominal symptoms (Martin‐Gallausiaux et al. 2021). This evidence suggests that the role of bacteria in the relationship between alcohol and IBS is still worth exploring.

In our study, psychiatric disorders, including depression (broad), MDD, and ADHD, were found to be explicit mediation factors. In both observational and experimental studies, high alcohol intake can lead to an increase in psychiatric disorders (Xu et al. 2022; Boden and Fergusson 2011). Studies have proved that alcohol use disorder and depression share a common genetic predisposition, and dysfunction of the reward and stress systems may be one of the mechanisms of action (McHugh and Weiss 2019).

IBS is a typical disorder of brain‐gut interactions (Mayer et al. 2023). Alcohol affects IBS through the gut‐brain axis via multisystem interactions, including neuroendocrine and immune pathways. Alcohol can modulate neurotransmitter activity. Prolonged alcohol consumption results in the development of tolerance to many of the GABAergic effects of alcohol, including its anxiolytic, sedative, motor incoordinating, and positive reinforcing effects (Koulentaki and Kouroumalis 2018). Alcohol can damage the intestinal barrier, allowing microbial metabolites to enter the bloodstream and trigger immune responses (Stärkel et al. 2018).

The relationships between psychiatric disorders and IBS are widely recognized, especially depression disorders. Clinical observations and research found brain connection alterations in IBS patients, including the salience network, the default mode network, the sensorimotor network, and the central autonomic network, showing the critical role of the brain in generating and maintaining IBS symptoms (Mayer et al. 2023). At the same time, a study based on the Chinese population indicated that IBS patients and depression disease patients share a common genetic predisposition. SYT8 rs3741231 G allele and SSPO rs12536873 TT genotype are associated with both IBS and depression, which of these variants are important in neurogenesis and neurotransmission (Zhu et al. 2020). Therefore, combined with experimental data, we believe that depression is a clear intermediary factor.

ADHD was found to be a positive mediation factor through the Sobel test, while MVMR analysis showed a negative result. ADHD and alcohol use disorder share common genetic, neurobiological, and neuropsychological associations (Luderer et al. 2021). Having ADHD‐related genes, regardless of ADHD status, elevates the risk of alcohol use disorder, with greater risk associated with more genes involved (Wimberley et al. 2020). In addition, children with ADHD are more likely to suffer from functional gastrointestinal diseases (Petropoulos et al. 2024). However, due to the potential for more internalizing problems (such as anxiety and depression) associated with ADHD, ADHD may be an upstream factor for depression. Therefore, in MVMR, after adjusting for depression, the results became insignificant, suggesting that it may not function as a mediating factor in the relationship between alcohol and IBS. Notably, our research suggests that ADHD may be an independent risk factor involved in the pathogenesis of IBS, which warrants further exploration.

There were several limitations. Firstly, the data used in our study were exclusively sourced from European populations, limiting the generalizability of our experimental findings to other populations. Although the GWAS data have achieved remarkable sample sizes, they might still encounter statistical underpowering for certain specific exposure‐outcome associations, particularly those with weaker effects and limited sample sizes. However, by employing various robustness tests, our results had been verified to have strong statistical power.

## 5 Conclusion

Our study suggested that alcohol intake had causal relationships with IBS from a genetic perspective. Depression (broad), MDD, and ADHD disorders might be mediating factors through the brain‐gut axis. The mediating role of gut microbiota requires alternative research approaches for further elucidation. Further explorations should also be conducted to investigate the underlying mechanisms of how alcohol contributes to the development of IBS. Our findings suggest that reducing alcohol consumption may help reduce the risk of developing IBS, particularly in individuals with psychiatric disorders.

## Author Contributions

**Ruiqing Yuan:** investigation (equal), methodology (equal), software (equal), writing – original draft (lead). **Yajing Zhou:** conceptualization (lead), data curation (equal), resources (equal), validation (equal). **Yujie Ren:** formal analysis (equal), methodology (lead), software (equal), visualization (equal). **Xiaohua Hou:** funding acquisition (equal), methodology (equal), resources (equal), supervision (equal), writing – review and editing (equal). **Siran Zhu:** conceptualization (equal), funding acquisition (equal), project administration (equal), resources (equal), supervision (equal), writing – review and editing (lead).

## Ethics Statement

All participants had given written informed consent in the original GWAS projects.

## Conflicts of Interest

The authors declare no conflicts of interest.

## Supporting information


**Figure S1:** Leave‐one‐out plot for alcohol intake frequency on IBS.

**Figure S2:** Funnel plot for alcohol intake frequency on IBS.

**Figure S3:** Leave‐one‐out plot for drinks per week on IBS.

**Figure S4:** Funnel plot for drinks per week on IBS.

**Table S1:** Datasets used in our analyses.
**Table S2:** Mendelian randomization results between IBS and gut microbiota features.
**Table S3:** Mendelian randomization results between gut microbiota features and IBS.
**Table S4:** Mendelian randomization results between alcohol intake frequency and gut microbiota features.
**Table S5:** Mendelian randomization results between psychiatric disorders and IBS.
**Table S6:** Mendelian randomization results between alcohol intake frequency and psychiatric disorders.
**Table S7:** Details of IVs utilized for alcohol intake frequency.
**Table S8:** Details of IVs utilized for drinks per week.

## Data Availability

The genetic data for this study are available from MRC‐IEU, GSCAN, Psychiatric Genomics Consortium, and MiBioGen. Detailed data of this study are shown in the Supporting Information and are available from the corresponding author upon reasonable request.
