# Supplementary figures and images for: Mendelian Randomization Analysis Reveals Causal Associations Between Beverages and Irritable Bowel Syndrome: Alcohol, but Not Others
Source: Food Sci Nutr. 2025 Jul 31;13(8):e70761. doi: 10.1002/fsn3.70761 (PMC12313541; doi:10.1002/fsn3.70761)

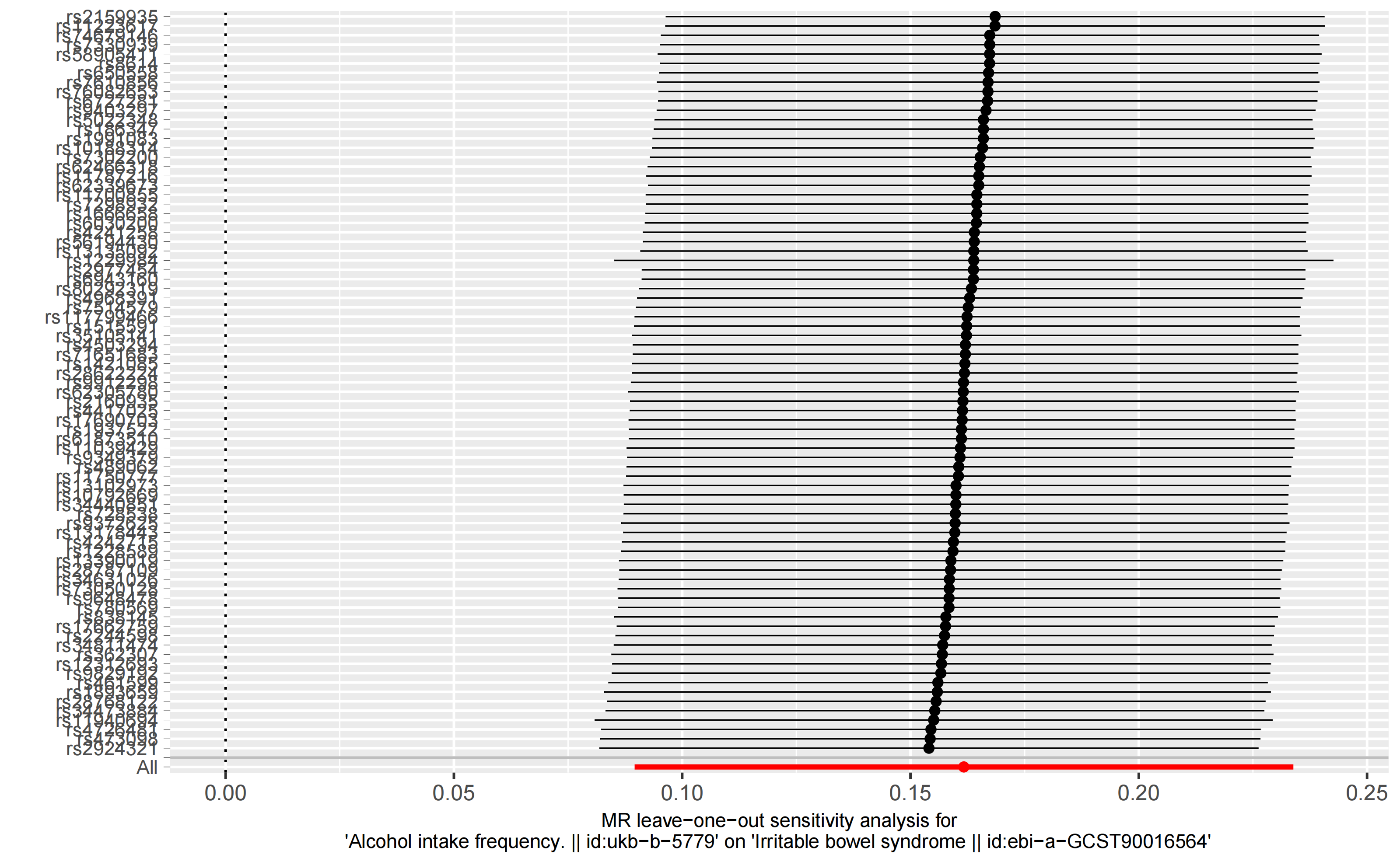

Supplement: Supplementary file 1 — Figure S1: Leave‐one‐out plot for alcohol intake frequency on IBS. [file FSN3-13-e70761-s005.tif]

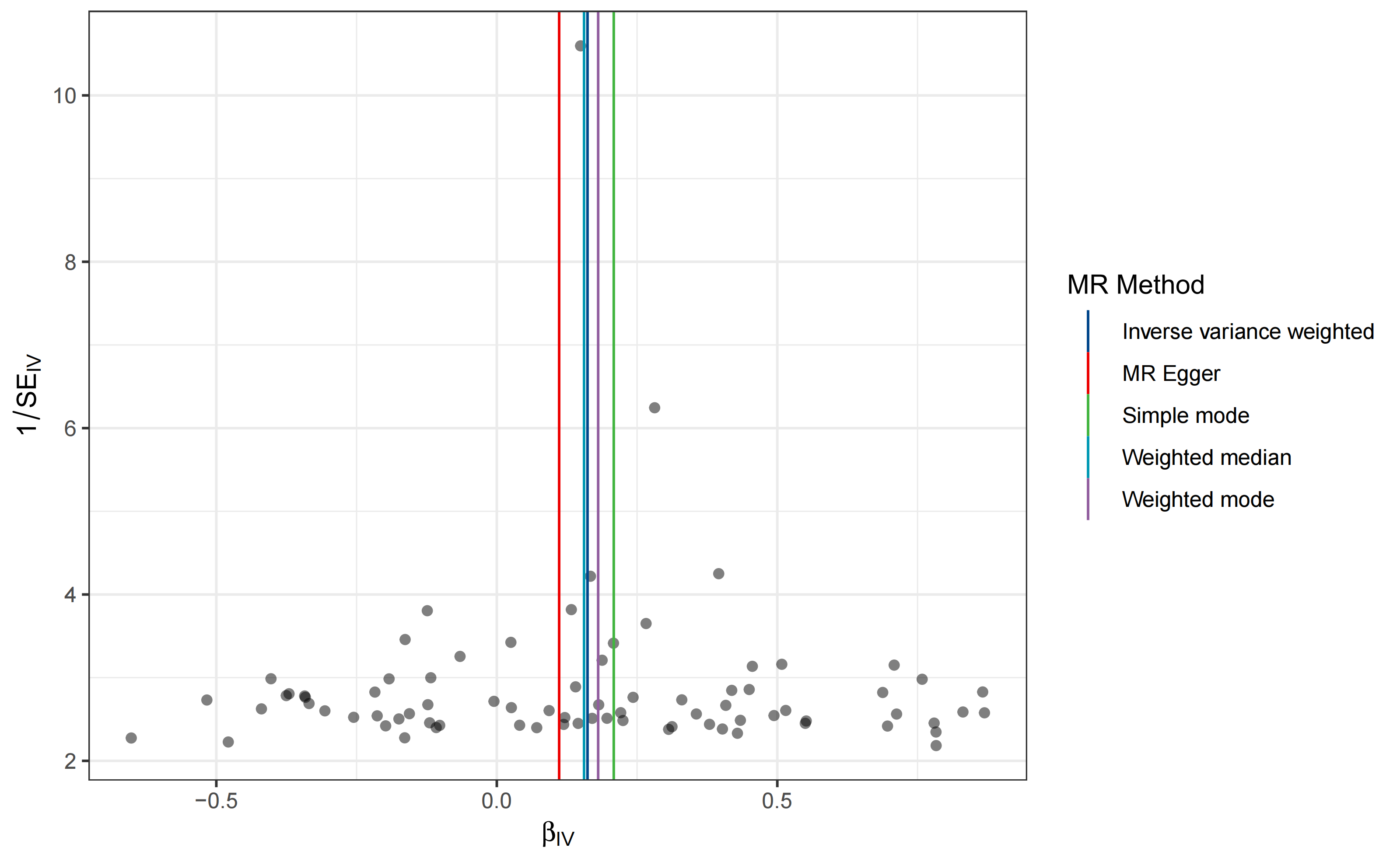

Supplement: Supplementary file 2 — Figure S2: Funnel plot for alcohol intake frequency on IBS. [file FSN3-13-e70761-s002.tif]

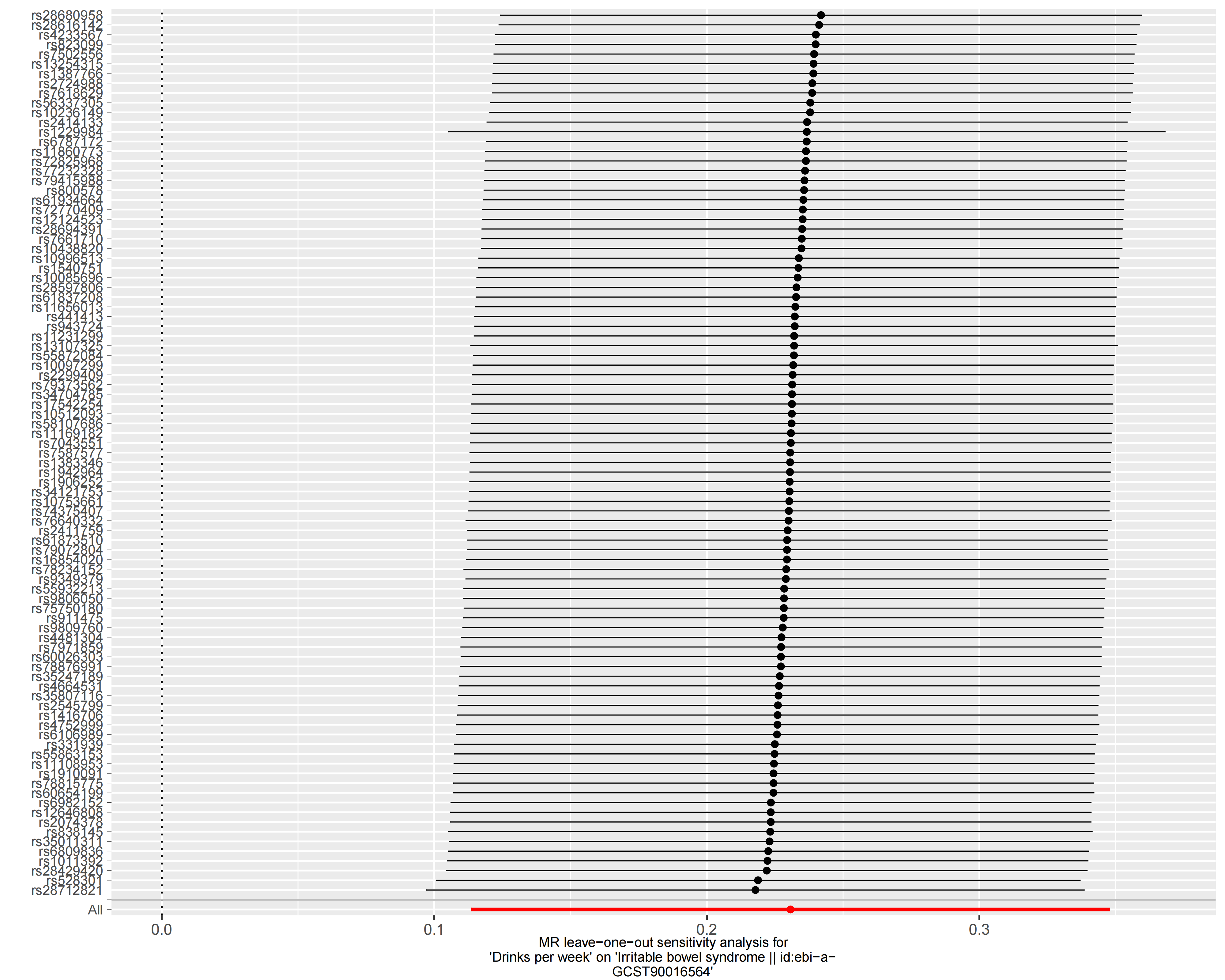

Supplement: Supplementary file 3 — Figure S3: Leave‐one‐out plot for drinks per week on IBS. [file FSN3-13-e70761-s004.tif]

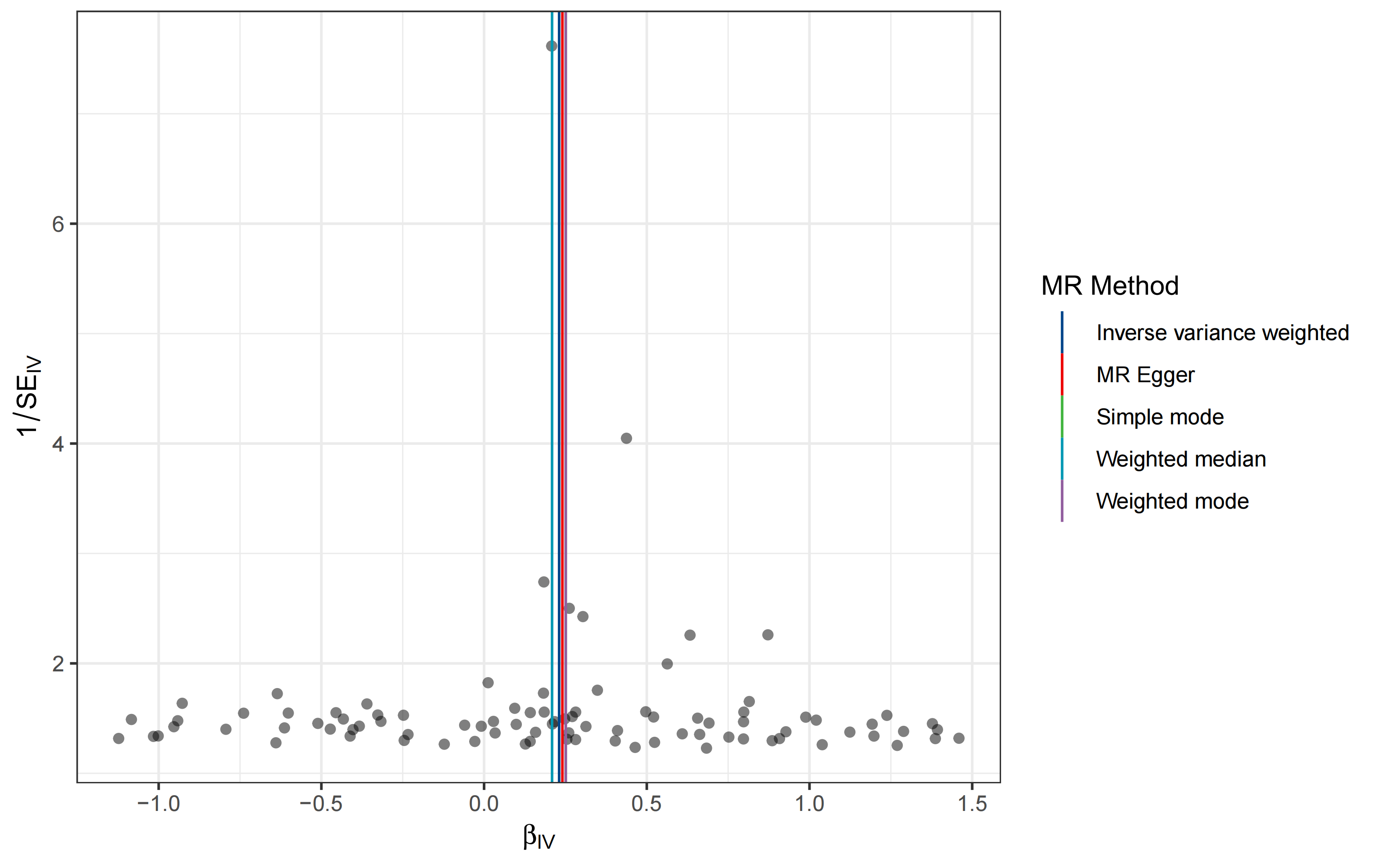

Supplement: Supplementary file 4 — Figure S4: Funnel plot for drinks per week on IBS. [file FSN3-13-e70761-s003.tif]
